# H3K4me3 CUT&Tag and Transcriptome Analysis Reveal the Epigenetic Regulatory Landscape in Mammary Gland Tissues of Yili Horses at Different Lactation Stages

**DOI:** 10.3390/ani16060891

**Published:** 2026-03-12

**Authors:** Lingling Liu, Hang Cao, Haiyu Ma, Bin Chen, Wujun Liu

**Affiliations:** College of Animal Science, Xinjiang Agricultural University, Urumqi 830052, Chinacaohang@xjau.edu.cn (H.C.); haiyu_ma@163.com (H.M.); chenbin_1998@163.com (B.C.)

**Keywords:** Yili horse, H3K4me3, mammary gland tissue, CUT&Tag, transcriptome

## Abstract

H3K4me3, a well-established histone modification associated with active promoters, plays a critical role in orchestrating gene expression programs that govern mammary gland development and lactation. In this study, we present the first comprehensive epigenomic profiling of H3K4me3 modifications during mammary gland development in Yili horses using Cleavage Under Targets and Tagmentation (CUT&Tag) and RNA sequencing. Mammary gland tissues were collected from two developmental stages—early lactation and peak lactation. A total of 393 differentially expressed genes (DEGs) were identified between two groups, among which 72 DEGs (54 upregulated H3K4me3 targets and 18 downregulated targets) were directly regulated by H3K4me3. KEGG enrichment analyses revealed that these DEGs were involved in ECM–receptor interaction, focal adhesion, the PI3K-Akt signaling pathway, and the calcium signaling pathway. In these pathways, five genes were identified as potential regulators of mammary gland development. Among these, PTGES, COL1A1, PDGFRB, and RYR1 exhibited consistent upregulation at both the transcriptomic and chromatin levels, whereas PRKAG3 showed significant downregulation. These findings offer novel insights into the epigenetic regulation of lactation in horses and lay a theoretical foundation for improving milk production traits through targeted molecular breeding strategies.

## Simple Summary

Understanding how genes are turned on or off during lactation can help improve milk production and animal health. In this study, we examined the activity of specific genes in the mammary glands of Yili horses at two important time points: early lactation and peak lactation. We focused on a molecular marker called H3K4me3, which is found near genes that are actively being used by the cell. By combining two advanced techniques that measure both gene activity and molecular markers, we discovered several genes that are strongly linked to milk production and immune response. One gene, PTGES, was especially active during peak lactation. These changes suggest that the mammary gland shifts its focus from immune defense to stable energy use as lactation progresses. This research provides valuable knowledge about how milk production is controlled at the genetic level and offers new opportunities to improve breeding strategies, enhance milk yield, and support the health of dairy animals.

## 1. Introduction

Mammary gland development and lactation function are central topics in dairy science research [1]. Understanding the transcriptional and epigenetic mechanisms that regulate mammary gland function is essential for elucidating how milk synthesis and secretion are coordinated in livestock. Recent studies show that mare’s milk has a distinct nutritional profile compared with other dairy species, characterized by lower fat but higher lactose, a greater proportion of whey proteins, and elevated levels of unsaturated fatty acids [2,3]. These unique biochemical features highlight the physiological specificity of equine lactation and underscore the need to better understand the regulatory mechanisms governing milk production in mares. In mammals, gene expression programs controlling lactation are governed by the interplay of transcription factors, cofactors, and cis-regulatory elements such as promoters and enhancers, which together orchestrate stage-specific transcriptional activation [4,5,6,7,8,9,10].

The Yili horse was developed in the 20th century by crossbreeding the Kazakh horse with Orlov Trotter, Don, Budyonny, and Akhal-Teke breeds [11]. It has since been established as an independent dairy-type breed in Xinjiang, China. The Yili horse is characterized by high milk yield, broad environmental adaptability, and remarkable tolerance to the region’s harsh continental climate, including severe cold and short pasture growth seasons [12]. Over the entire lactation period, the average daily milk yield of the Yili horse is 9.20 kg, with a milk protein content of 1.57% and a milk fat content of 0.75% [13]. In mares, milk is synthesized primarily within secretory alveoli composed of epithelial cells, which are surrounded by contractile myoepithelial cells and embedded in a connective tissue matrix rich in blood vessels that supply nutrients and support milk ejection [14,15]. Lactation in mares exhibits distinct stage-related dynamics, with milk yield typically peaking around 30–60 days postpartum, reflecting a physiological transition from the initial phase of secretory activation to a production-stabilized, high-yield stage [16]. Previous transcriptomic studies on equine mammary glands are limited but have revealed several common features. In Kazakh horses, differentially expressed genes related to lipid metabolism, protein synthesis, and immune regulation were identified between mares with different milk yields [17]. Studies comparing pregnant and non-pregnant mares further showed that pathways such as steroid hormone biosynthesis, ECM–receptor interaction, MAPK and PI3K–Akt signaling are involved in mammary activation and tissue remodeling before lactation [18,19]. In Mongolian mares, cell proliferation, metabolic, and immune pathways were also enriched during lactation [20]. Together, these findings indicate that hormonal regulation, extracellular matrix remodeling, and metabolic activation are key transcriptional features of equine mammary gland development, although dynamic transcriptomic changes across distinct lactation stages remain unexplored in the Yili horse.

Chromatin modifications, especially histone methylation, play key roles in defining promoter activity and transcriptional competence [21,22,23]. Among them, trimethylation of histone H3 at lysine 4 (H3K4me3) marks active promoters and is strongly correlated with gene activation [24,25]. Profiling of H3K4me3 in cattle has revealed promoter-specific variation associated with milk production traits [26], while in pigs, chicken and fish, dynamic H3K4me3 remodeling has been linked to developmental and environmental adaptation [27,28,29]. Nevertheless, little is known about H3K4me3 distribution and function in the mammary glands of horses. To address this gap, we applied Cleavage Under Targets and Tagmentation (CUT&Tag), a high-resolution chromatin profiling method requiring minimal input and offering superior signal-to-noise ratio compared with conventional ChIP-seq [30,31]. This approach enables precise mapping of histone modifications at promoter regions, thereby identifying regulatory elements that correlate with transcriptional activity [32]. This technique has been successfully applied to profile transcription factors and chromatin modifications in various animal species, with most studies focusing on pigs and cattle [33,34,35].

The aim of this study was to characterize genome-wide H3K4me3 dynamics and their transcriptional consequences in the mammary gland of Yili horses during early and peak lactation stages.

## 2. Materials and Methods

### 2.1. Sample Collection

A total of six 8-year-old healthy Yili horses, numbered M1_RXL, M2_RXL, M3_RXL, M4_RXL, M5_RXL, and M6_RXL, were obtained from a farm in Zhaosu County, Yili Kazakh Autonomous Prefecture, Xinjiang Uygur Autonomous Region, China (latitude 43°09′–43°15′ N, longitude 80°08′–81°30′ E). To ensure a structured and balanced experimental design, the horses were divided into two groups according to their lactation stage: three in early lactation (month 1; sample names: M1–M3; group S1) and three in peak lactation (month 3; sample names: M4–M6; group S2); RXL denotes that mammary tissue was consistently collected from the left mammary gland of horses. All mares were in their fourth parity, and each delivered a single foal at the parturition preceding tissue collection. The experimental individuals were grazed on a fixed pasture and, during lactation, each received 1 kg of concentrate feed daily (Junli Agro-Tech Co., Ltd., Zhaosu County, Yili Kazakh Autonomous Prefecture, Xinjiang, China).

Mammary gland tissues were collected post slaughter from the same anatomical location in each horse. All horses had clinically healthy mammary glands, with no signs of mastitis confirmed by visual inspection and palpation. Tissues were collected at approximately 12:00 noon following an overnight fast. The slaughter procedures followed industry standards and ethical guidelines. During sampling, the mammary gland was transversely sectioned at the midline, and approximately 10 g of glandular tissue was collected from the central region of the half away from the teat. The collected tissue was immediately placed into RNase-free tubes (Biosharp Biotech Co., Ltd., Hefei, Anhui, China), snap-frozen in liquid nitrogen, and subsequently stored at −80 °C for long-term preservation until further RNA-seq and CUT&Tag (H3K4me3) analysis.

### 2.2. Transcriptome Sequencing

Total RNA was extracted from tissue samples using TRIzol™ reagent (Thermo Fisher Scientific, Waltham, MA, USA), and RNA integrity was assessed with the Qsep400 system (BiOptic, La Canada Flintridge, CA, USA). RNA sequencing (RNA-seq) libraries were constructed from 1 μg of total RNA using the VAHTS Universal V10 Library Prep Kit for Illumina (Cat. No. NR606; Vazyme Biotech Co., Ltd., Nanjing, Jiangsu, China) [36], following the manufacturer’s instructions. The library preparation process included poly(A) mRNA enrichment, RNA fragmentation, first-strand cDNA synthesis with random hexamer primers, second-strand synthesis, end repair, A-tailing, adapter ligation, and PCR amplification. Paired-end sequencing (150 bp) was performed on the Illumina NovaSeq 6000 platform (Illumina Inc., San Diego, CA, USA).

Using Cutadapt (version 1.11) (https://cutadapt.readthedocs.io/, accessed on 17 March 2025), the raw sequencing reads were subjected to adapter and low-quality read removal (reads with more than 20% of bases having Q value ≤ 20 (where the Q value represents the base-calling error probability, with Q20 corresponding to a 1% error rate) or an ambiguous sequence content (“N”) exceeding 5%), and paired reads with >10% unidentified nucleotides (ratio in one read) were filtered to obtain clean data. Clean reads were then aligned to the Equus caballus reference genome (EquCab3.0) using HISAT2 (version 2.1.0), allowing a maximum of two mismatches [37]. Gene annotations were obtained from the NCBI non-redundant (NR) protein database. Transcript abundance was quantified using featureCounts (version 1.6.0) and normalized to fragments per kilobase of transcript per million mapped reads (FPKM) [38]. Functional enrichment analyses of DEGs were conducted with the R package clusterProfiler [39] for Gene Ontology (GO; http://geneontology.org/, accessed on 17 March 2025) [40] and Kyoto Encyclopedia of Genes and Genomes (KEGG; http://www.genome.jp/kegg/, accessed on 17 March 2025) [41] pathways, with statistical significance determined using hypergeometric testing at *p* < 0.05.

### 2.3. H3K4me3 CUT&Tag Analysis

CUT&Tag assays were conducted by Wuhan IGENEBOOK Biotechnology Co., Ltd. (Wuhan, China) (http://www.igenebook.com, accessed on 17 March 2025) following previously described protocols [30]. The CUT&Tag assays were performed on nuclei isolated from the same fresh mammary tissue biopsies used for RNA-seq analysis. Briefly, approximately 200–400 mg of fresh tissue was finely minced and subjected to cell disruption in 1 mL of lysis buffer at 37 °C. The resulting suspension was filtered through 40 µm cell strainers to remove debris. After washing, the nuclei were counted, and aliquots containing ~50,000 nuclei were prepared for each CUT&Tag reaction. The cell population was incubated with concanavalin A-coated magnetic beads for 15 min at room temperature. The bead-bound cells were then resuspended and incubated overnight at 4 °C with the appropriate primary antibody (Cat. No. 9751; Cell Signaling Technology, Danvers, MA, USA) or IgG control (Cat. No. 2729S; Cell Signaling Technology, Danvers, MA, USA). Subsequently, goat anti-rabbit secondary antibody (Cat. No. ab207-01; Vazyme Biotech Co., Ltd., Nanjing, Jiangsu, China) was added and incubated for 30 min at room temperature, followed by magnetic separation to remove unbound antibodies. The pA–Tn5 adapter complex was then added and incubated at room temperature for 1 h, and unbound pA–Tn5 was removed. Next, cells were resuspended in tagmentation buffer and incubated at 37 °C for 1 h to perform tagmentation, after which immunoprecipitated DNA was collected.

For library amplification, 21 µL of DNA (~40 ng/μL) was mixed with 2 µL of a universal i5 primer and a uniquely barcoded i7 primer (each sample was assigned a distinct barcode, 4 µM). Primers were provided as part of the VAHTS Universal Adaptors Set for Illumina (Vazyme, Cat. No. TD203, Nanjing, China). A total of 25 µL of NEBNext HiFi 2× PCR Master Mix (Cat. No. M0541, NEB, Ipswich, MA, USA) was added and thoroughly mixed. PCR amplification was performed using the following thermocycling conditions: 72 °C for 5 min (gap filling), 98 °C for 30 s, followed by 14 cycles of 98 °C for 10 s and 63 °C for 30 s, and a final extension at 72 °C for 1 min before holding at 8 °C. The resulting libraries were sequenced on the Illumina NovaSeq 6000 platform using the PE150 strategy.

Raw sequencing reads were filtered to remove low-quality bases using Trimmomatic (version 0.36) [42] under the following parameters: ILLUMINACLIP: adapters.fa:2:30:10; LEADING:3; TRAILING:3; SLIDINGWINDOW:4:15; and MINLEN:20. Clean reads were aligned to the EquCab3.0 reference genome using BWA (version 0.7.15) [43], and PCR duplicates were removed with Samtools (version 1.3.1) [44]. DeepTools (v2.5.4) was employed to quantify the distribution of reads ±2 kb around the transcription start sites (TSSs) from the aligned BAM files and to generate the corresponding signal fraction profiles [45]. Peak calling was performed with MACS2 (version 2.1.2) using default parameters (bandwidth: 300 bp; model fold: 5, 50; *p*-value: 0.00001). MACS2 estimates a local background (λ_local) for each genomic position using multiple window sizes and compares the observed read counts with the expected background counts. The significance of each candidate peak is tested using a Poisson distribution, and peaks with *p*-value < 1 × 10^−5^ were considered significantly enriched. Peaks with summits located closest to transcription start sites (TSSs) were assigned to the corresponding genes [46]. Using the DiffBind package (v1.16.3) (https://bioconductor.org/packages/DiffBind, accessed on 5 April 2025), reads overlapping each peak were counted and the differential peak between comparison groups was assessed to identify differentially modified regions. Motif enrichment analysis within peaks was performed using HOMER (version 4.11) [47]. Known transcription factor binding motifs were identified using default parameters, and enrichment significance was calculated relative to background regions. GO and KEGG pathway enrichment analyses were conducted using the same hypergeometric test method applied in RNA-seq analysis, with a significance threshold of *p* < 0.05.

### 2.4. Integrated Analysis of CUT&Tag and RNA-Seq Data

We performed an overlap analysis between CUT&Tag H3K4me3 target genes and differentially expressed genes (DEGs) identified by RNA-seq using Evenn (https://www.bic.ac.cn/test/venn/#/, accessed on 9 September 2025). GO and KEGG enrichment analyses were subsequently conducted on the overlapping gene set to explore their functional and pathway associations. GO and KEGG enrichment analyses of the overlap genes were conducted using DAVID (https://davidbioinformatics.nih.gov/summary.jsp, accessed on 9 September 2025).

### 2.5. Statistical Analysis

Differential gene expression analysis was performed using DESeq2, with |log2FC| > 1 and *p*-value < 0.05 considered significant. Differential H3K4me3 peaks were identified using MACS2 with a cutoff of *p* < 1 × 10^−5^. GO and KEGG enrichment analyses were conducted in DAVID, and pathways with *p* < 0.05 were treated as significantly enriched. Data visualization and statistical summaries were generated using R software (version 4.5.0).

## 3. Results

### 3.1. Transcriptome Sequencing Quality Control and Alignment Results

Following quality filtering and removal of low-quality raw reads, a total of 131,446,112 clean reads were obtained for the S1 group and 126,304,430 for the S2 group. Each sample generated approximately 63.43 GB of clean bases. These clean reads were aligned to the Equus caballus reference genome, achieving a mapping efficiency of 98.06%, with over 91.81% of reads mapping uniquely to the genome. Across all samples, more than 96.82% of bases attained a Q30 quality score, and the average GC content was 50.15% (Table S1), thereby confirming the high quality of the sequencing data and its suitability for downstream alignment and analytical workflows.

**Figure 1:**
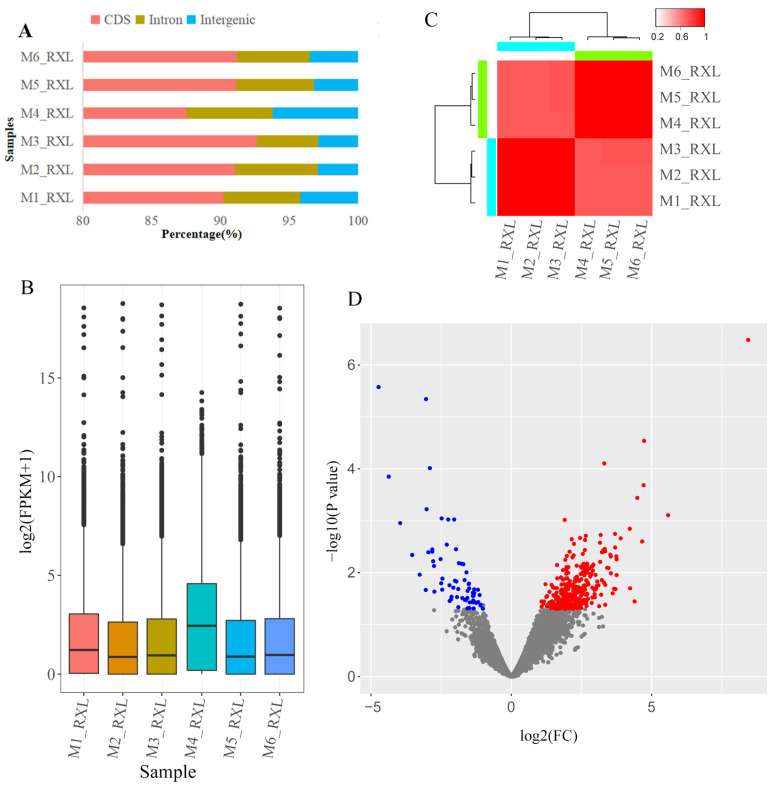
Transcriptome analysis of mammary gland tissues in horses at different lactation stages. (**A**) Distribution of sequencing reads across genomic features. (**B**) Gene expression levels across six samples. Bars represent the mean expression using FPKM quantitative expression for each lactation stage. The Y-axis is log2-transformed, and a pseudocount of 1 was added to include genes with zero expression. Error bars indicate the Standard Error of the Mean (SEM). Individual dots represent the FPKM values for each gene. (**C**) Correlation heatmap plot of all six samples based on the expression genes. Samples from early lactation samples (M1_RXL, M2_RXL, M3_RXL) and peak lactation samples (M4_RXL, M5_RXL, M6_RXL) showed higher internal correlations. (**D**) Volcano plot showing the DEGs in the S2 group compared with the S1 group, with red representing upregulated genes and blue representing downregulated genes.

To assess the quality of our RNA-seq libraries, we analyzed the distribution of aligned reads across different genomic features (Figure 1A). For five samples (M1_RXL, M2_RXL, M3_RXL, M5_RXL, M6_RXL), a high and consistent proportion of reads mapped to protein-coding sequences (CDSs), averaging 91.26%, while a very low percentage was found in intronic (avg. 5.39%) and intergenic regions (avg. 3.35%). Sample M4_RXL showed a slightly different pattern, with a lower proportion of reads in CDS regions (87.52%) and a higher proportion in intronic (6.25%) and intergenic regions (6.23%). The overall gene expression abundance was consistent across all six samples, with some variation between individual samples (Figure 1B). Although M4 showed a modest global shift in log2(FPKM+1), it clustered with peak-lactation samples in the correlation heatmap, indicating it is not an expression outlier. Correlation analysis of early lactation samples (M1_RXL, M2_RXL, M3_RXL) and peak lactation samples (M4_RXL, M5_RXL, M6_RXL) showed high intragroup concordance and clear intergroup divergence (Figure 1C), indicating the suitability of the data for differential gene expression analysis. A total of 393 DEGs were identified, of which 326 were upregulated and 67 were downregulated (Figure 1C, Table S2).

**Figure 2:**
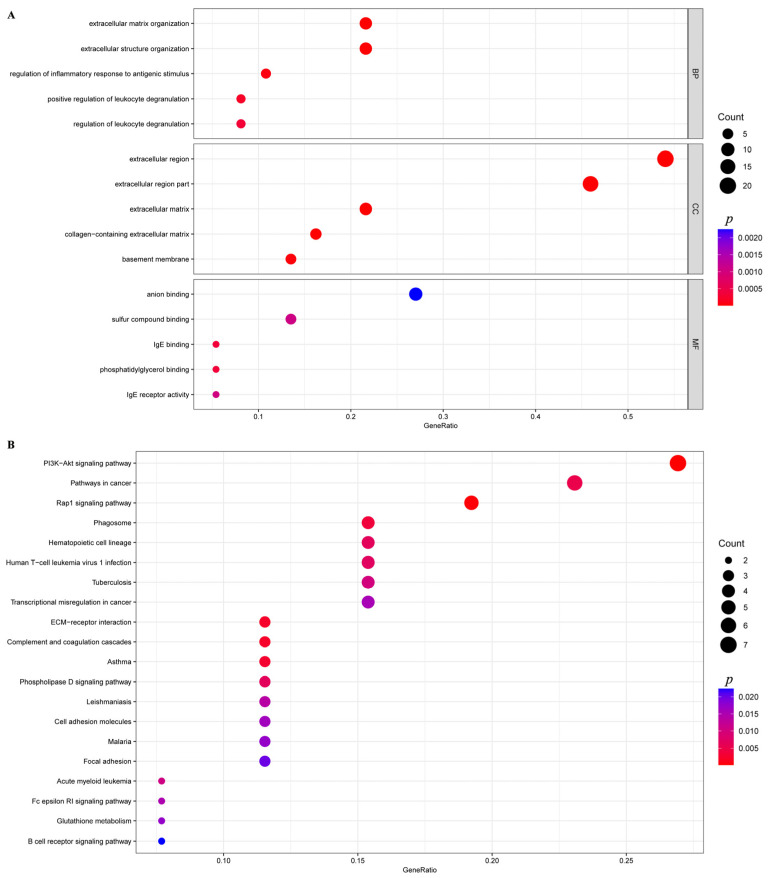
GO and KEGG of DEGs. (**A**) GO enrichment analysis of DEGs. (**B**) KEGG pathway enrichment analysis. *p* values are unadjusted. GeneRatio indicates the proportion of genes within the target gene set that are annotated to a given pathway or functional module, calculated as the number of such genes divided by the total number of genes in the target set.

GO and KEGG enrichment analysis was performed specifically on the 326 upregulated genes. These genes were annotated into 468 subcategories across the three major GO functional classifications, including 341 biological process (BP), 52 cellular component (CC), and 75 molecular function (MF) categories. The top 20 most significantly enriched three terms are detailed in Figure 2A. Notably, the extracellular matrix (GO:0031012) in the CC category and extracellular matrix organization (GO:0030198) in the BP category were significantly enriched (Table S3).

Based on DEG annotations, KEGG pathway analysis provided deeper insights into the biological processes underlying the peak lactation period in Yili horse. Compared with the S1 group, DEGs were mainly enriched in pathways related to “signal transduction,” “cell adhesion and ECM remodeling,” “immune response and infection,” “cancer,” and “metabolism” (Figure 2B). Among these, five pathways were nominally enriched with unadjusted *p* values < 0.05: the PI3K-Akt signaling pathway (04151), ECM–receptor interaction (04512), focal adhesion (04510), the Wnt signaling pathway (04310), and the Rap1 signaling pathway (04015) (Table S4).

### 3.2. H3K4me3 Modification Changes in Two Stages of Yili Horse

**Figure 3:**
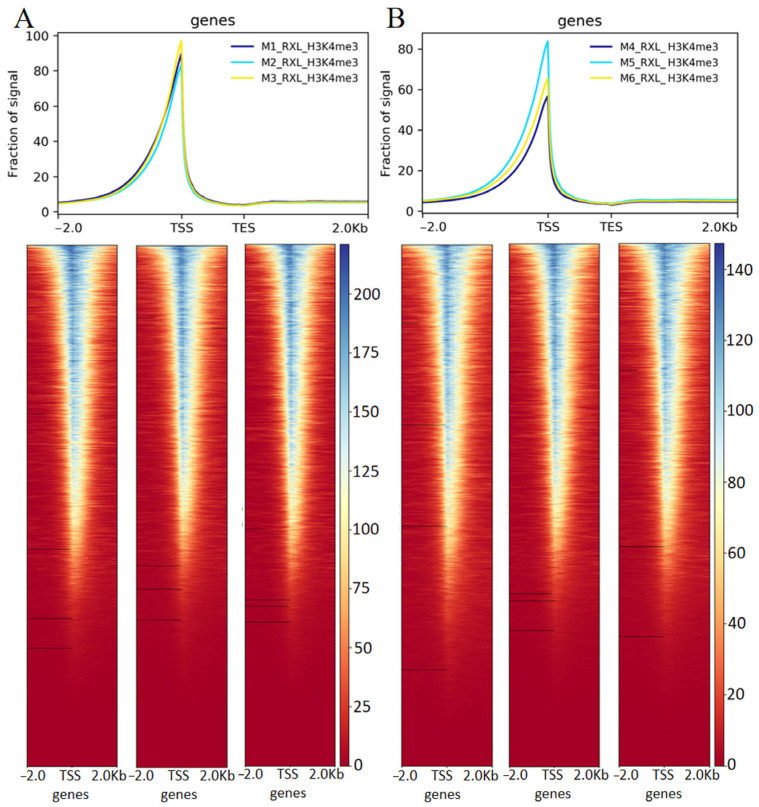
Genome-wide CUT&Tag analysis of histone H3K4me3. (**A**) Distribution of reads across genomic regions in the S1 group. (**B**) Distribution of reads across genomic regions in the S2 group. The fraction of the signal is calculated as the ratio of the signal intensity in the H3K4me3 sample to the signal intensity in the control antibody sample.

**Figure 4:**
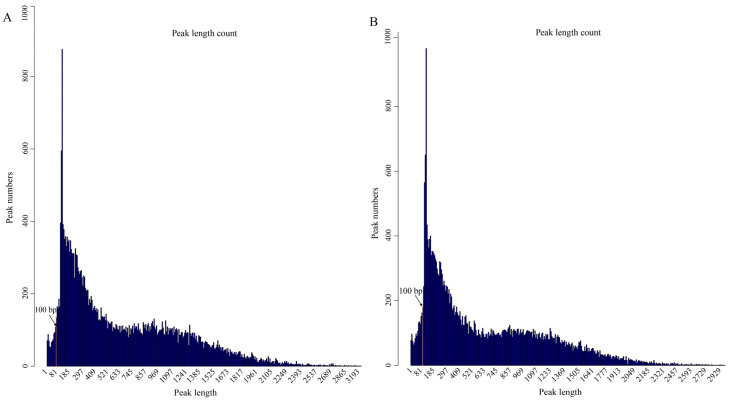
Peak length distribution in two groups. (**A**) Peak length distribution in the S1 group. (**B**) Peak length distribution in the S2 group. Peak length indicates the distance between the start and end of the peak.

In this study, mammary gland tissues from two lactation stages were collected to investigate the role of H3K4me3 histone modification during peak lactation in Yili horses using CUT&Tag. Following cell preprocessing and library quality assessment, sequencing was performed on an Illumina platform, yielding a total of 193,674,036 clean reads across six samples, with an average Q30 of 94.6% and an average mapping rate of 99.43% (Table 1). H3K4me3 signals were strongly enriched near transcription start sites (TSSs), while enrichment outside these regions was minimal (Figure 3A,B). The signal from the control IgG antibody was low and uniform across the genome (Figure S1), supporting the specificity of the H3K4me3 enrichment shown in Figure 3. We examined the length distribution of H3K4me3-enriched peaks identified by CUT&Tag in the S1 and S2 groups. As expected for a high-resolution epigenomic profiling method, the vast majority of peaks were narrow: approximately 80% ranged from 1 to 1000 bp in length, with the highest density observed between 100 and 300 bp (Figure 4A,B). Notably, the S2 group exhibited a greater number of very short peaks centered around 100 bp compared to S1.

**Figure 5:**
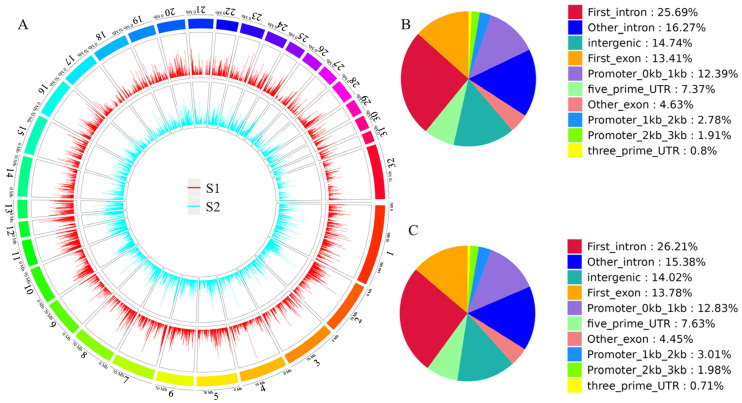
Genomic localization and functional annotation of H3K4me3 peaks. (**A**) Genome-wide distribution of peaks in the S1 and S2 groups. The outermost ring displays chromosomes 1–31 (autosomes) and chromosome 32 (X chromosome) of the EquCab3.0 reference genome, and the inner two tracks represent H3K4me3 signal intensity in the S1 (red) and S2 (blue) groups, respectively. (**B**) Proportion of peaks across different functional genomic regions in the S1 group. (**C**) Proportion of peaks across different functional genomic regions in the S2 group.

H3K4me3 peaks identified across different lactation stages were distributed across all chromosomes of the horse genome (Figure 5A). The genomic locations of the peaks were classified into eight categories: intergenic, intron, exon, 3′ UTR, 5′ UTR, promoter (≤1 kb), promoter (1–2 kb), and promoter (2–3 kb). H3K4me3 signals were predominantly enriched in the first intron, intergenic regions, first exon, and promoter (≤1 kb) categories in both S1 and S2 groups (Figure 5B,C).

A total of 32,457 and 31,644 peaks were identified in the S1 and S2 groups, respectively. Computational analysis revealed 4146 upregulated and 4959 downregulated H3K4me3 peaks (*p* < 0.00001) (Tables S5 and S6). Annotation of these differential peaks identified 2352 known upregulated genes and 2595 known downregulated genes (Tables S5 and S6).

**Figure 6:**
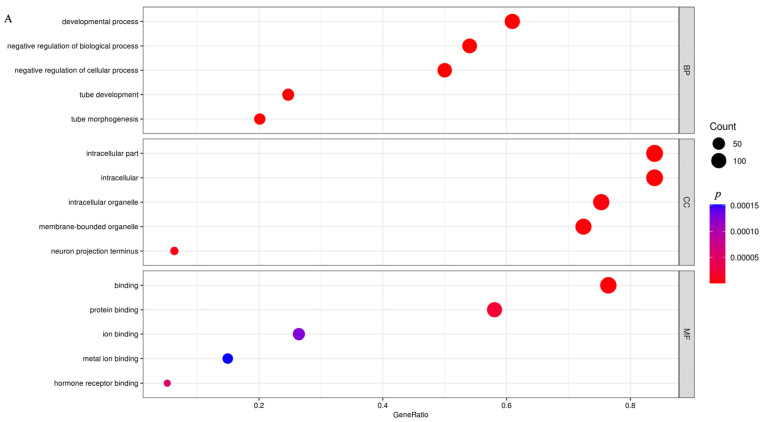

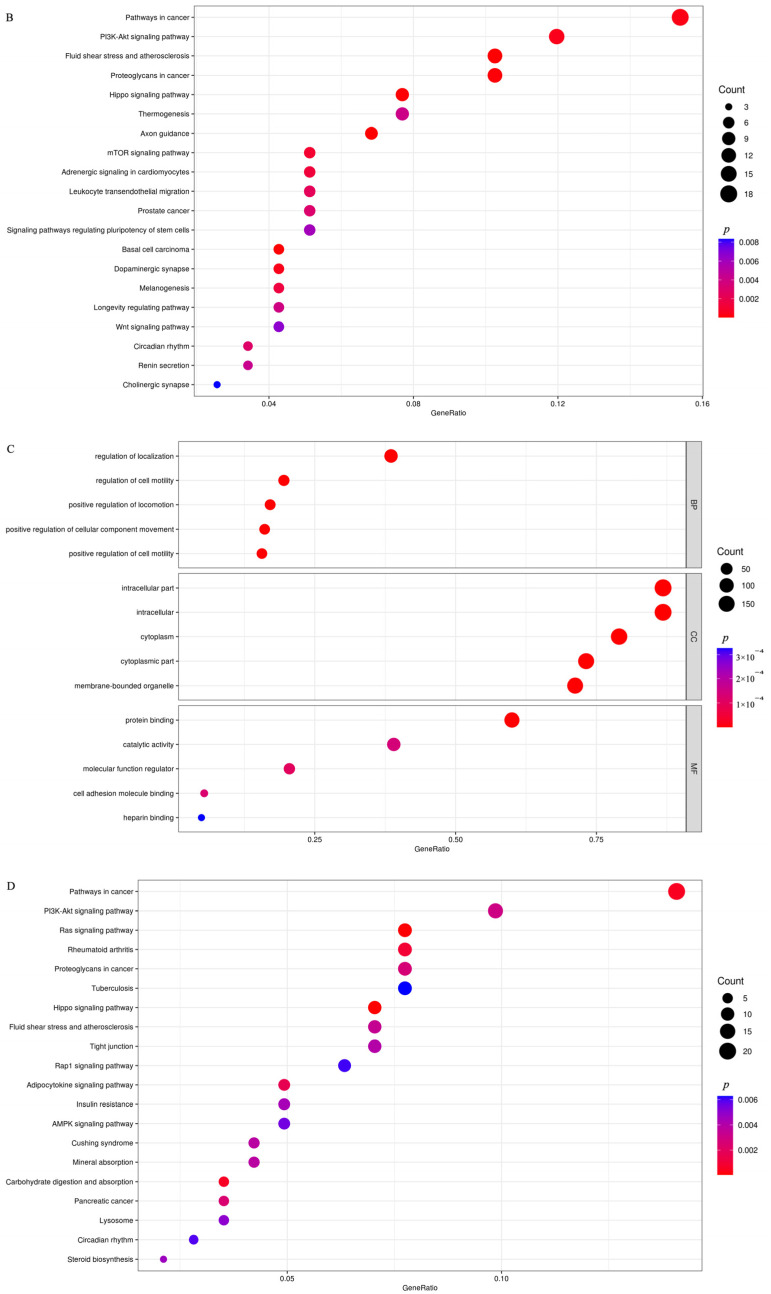
Functional enrichment and motif analysis of differential H3K4me3 targets. (**A**) Gene Ontology (GO) enrichment of upregulated genes. (**B**) KEGG pathway enrichment of upregulated genes. (**C**) GO enrichment of downregulated genes. (**D**) KEGG pathway enrichment of downregulated genes.

**Figure 7:**
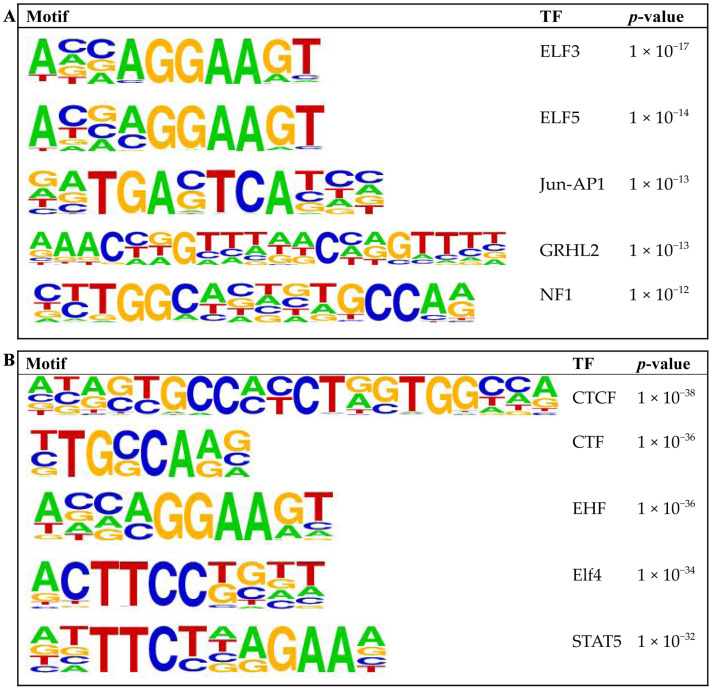
Enriched transcription factor motifs in upregulated and downregulated H3K4me3 peaks. (**A**) Top five enriched known motifs of upregulated peak-associated genes. (**B**) Top five enriched known motifs of downregulated peak-associated genes.

GO and KEGG pathway enrichment analyses showed that upregulated genes were mainly involved in developmental processes, tube development, and negative regulation of biological processes, with enriched pathways including the PI3K-Akt signaling pathway, Hippo signaling pathway, and axon guidance (Figure 6A,B, Table S7). Downregulated genes were primarily enriched in positive regulation of locomotion, intracellular components, and intracellular parts, with enriched pathways including rheumatoid arthritis, the Ras signaling pathway, and the carbohydrate digestion and absorption pathway (Figure 6C,D, Table S8). Figure 7A,B show the top five binding motifs for H3K4me3 upregulated and downregulated targets, respectively. The total number of significant binding motifs was 128 for upregulated targets and 232 for downregulated targets, with enrichment significance determined by *p*-value (Table S9).

### 3.3. Integrative Analysis of Transcriptome and H3K4me3 CUT&Tag Data

**Figure 8:**
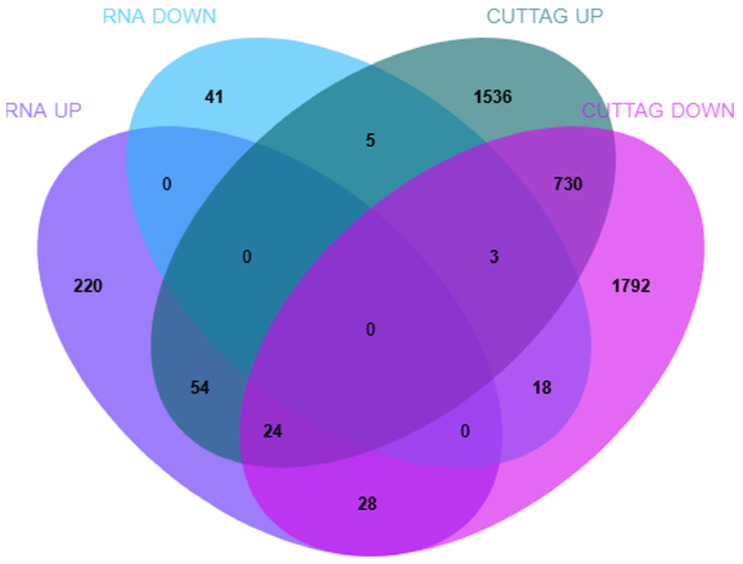
Venn diagram of DEGs according to RNA-seq analysis and CUT&Tag analysis. RNA UP/DOWN denotes up/downregulated DEGs, and CUT&Tag UP/DOWN denotes genes linked to increased/decreased H3K4me3 peaks. Discordant overlaps (RNA UP & CUT&Tag DOWN; RNA DOWN & CUT&Tag UP) may reflect temporal decoupling, alternative promoter usage, or post-transcriptional regulation. The overlap between CUT&Tag UP and CUT&Tag DOWN occurs at the gene level, as multiple differential peaks can map to the same gene with opposite directions.

The genome-wide binding sites of H3K4me3 in Yili horses were characterized using CUT&Tag. However, this alone is insufficient to fully elucidate the regulatory landscape of H3K4me3. Therefore, we performed an integrative analysis combining CUT&Tag and RNA-seq data to investigate the relationship between H3K4me3 occupancy and target gene expression. This analysis identified 54 upregulated and 18 downregulated H3K4me3 target genes, determined by overlapping up- and downregulated DEGs from RNA-seq with genes showing increased or decreased H3K4me3 signals from CUT&Tag, respectively (Figure 8, Tables S10 and S11).

The enriched GO terms of the identified genes were mainly associated with extracellular matrix organization, cell surface and membrane-associated components, and calcium- and growth factor-related molecular functions (Figure S2). KEGG enrichment analysis showed that the overlap genes were predominantly enriched in pathways related to extracellular matrix interaction and intracellular signaling, including ECM–receptor interaction, the apelin signaling pathway, PI3K–Akt signaling, and the calcium signaling pathway (Figure S3).

## 4. Discussion

To our knowledge, this is the first study to profile genome-wide H3K4me3 in the mammary gland of Yili horses using CUT&Tag integrated with RNA-seq. Our integrated CUT&Tag and RNA-seq analyses provide the first genome-wide view of H3K4me3 dynamics and their transcriptional correlates in Yili horse mammary tissue across two lactation stages. The sequencing data were of high technical quality (RNA-seq: Q30 > 96% mapping; CUT&Tag: Q30 94.6% mapping). At the chromatin level, H3K4me3 formed narrow, promoter-centered peaks and exhibited the strongest enrichment near transcription start sites (TSSs). This pattern aligns with the canonical role of H3K4me3 as a promoter-associated histone modification that marks transcriptional competence and initiation [48]. The localization of most H3K4me3-enriched nucleosomes within sharp, narrow peaks around TSSs validates both the reliability of the CUT&Tag assay and the expected promoter-centered distribution of this epigenetic mark [49]. Promoter-centered H3K4me3 around TSS is conserved across mammals and is reported in mouse mammary tissue during pregnancy/lactation [50]. Mouse studies further show lactation-specific distal enhancers, supporting broad regulatory reprogramming during lactation [51]. Given limited equid mammary epigenome resources, our results likely capture conserved lactation programs, while equid/breed specificity of individual loci awaits further validation [20].

### 4.1. Stage-Specific H3K4me3 Dynamics and Their Biological Implications

Building on this genome-wide characterization, comparative analysis between the two lactation stages revealed substantial remodeling of the H3K4me3 landscape, with approximately 32,000 peaks identified in each stage (S1: 32,457; S2: 31,644). Among these, 4146 peaks increased and 4959 decreased significantly (*p* < 0.00001). Peak annotation indicated that most signals were located in first introns, first exons, and so on, reinforcing the promoter-centric regulatory role of H3K4me3. This heightened promoter marking may reflect a transcriptionally “primed” chromatin state, facilitating rapid gene activation during the onset of lactation [52]. As the mammary gland transitions to peak lactation, regulatory programs may shift toward fewer but more stable, highly expressed genes, accompanied by a relative decrease in promoter H3K4me3 levels. Alternatively, gene expression in this stage may increasingly depend on distal enhancer activity and H3K27ac-mediated regulation [48,53], indicating a shift from promoter priming to enhancer-driven transcriptional maintenance.

### 4.2. Pathways Implicated in Lactation Biology

To further understand the functional impact of these chromatin changes, KEGG enrichment of RNA-seq data revealed significant involvement of pathways related to signal transduction, ECM remodeling and cell adhesion, immune responses, metabolism, and cancer-associated signaling. Five pathways were significantly enriched: PI3K–Akt signaling, ECM–receptor interaction, focal adhesion, Wnt signaling, and Rap1 signaling. The PI3K–Akt pathway is known to regulate epithelial cell survival, proliferation, and metabolic reprogramming, activating mTOR-dependent signaling required for protein and lipid synthesis in milk production [54,55]. ECM signaling plays a central role in mammary gland development by directing epithelial cell migration and acinar morphogenesis [56]. Wnt signaling regulates cell fate, proliferation, differentiation, and stem cell renewal, and its activity has been linked to multiple stages of mammary gland growth and involution [57,58]. Rap1 signaling contributes to epithelial polarity and the formation of hollow tubular structures, a key prerequisite for ductal and alveolar morphogenesis during lactation [59,60]. Integrating these transcriptional insights with epigenomic data revealed coherent biological themes associated with mammary development and lactation.

During the peak lactation, H3K4me3-upregulated genes were enriched in the PI3K–Akt signaling pathway, proteoglycans in cancer, Hippo signaling, and axon guidance. Consistently, RNA-seq analysis also highlighted significant enrichment of the PI3K–Akt pathway, indicating that this signaling pathway plays an important role during peak lactation in Yili mares. Previous studies have shown that the Hippo pathway regulates mammary epithelial differentiation and growth, and that its key effector TAZ influences mammary gland morphogenesis and governs the onset of mammary development [61,62,63].

Conversely, downregulated genes were enriched in oxytocin signaling, cellular senescence, the Ras signaling pathway, and the carbohydrate digestion and absorption pathway. The reduced oxytocin signaling during peak lactation may reflect feedback inhibition or decreased receptor sensitivity as lactation stabilizes [64], and its signaling tends to be transient rather than continuously upregulated at the transcriptional level. Decreased Ras signaling may indicate a shift away from proliferative and growth-related programs, as the mammary gland transitions from structural expansion to functional specialization at peak lactation. Much evidence indicates that oxytocin primarily regulates milk ejection rather than sustained milk synthesis. Yukinaga et al. reported that milk ejection remains the main physiological role for oxytocin in the mammary gland [65]. Together, these patterns suggest that during peak lactation, pathways related to structural growth, systemic metabolism, and transient neuroendocrine responses are toned down, while the mammary gland prioritizes epithelial secretory function and lactation-specific signaling.

GO and KEGG enrichment analyses of the overlap genes revealed significant enrichment in ECM–receptor interaction, Apelin signaling, PI3K–Akt signaling, and calcium signaling pathways. Notably, previous studies have shown that ECM–receptor interaction and PI3K–Akt signaling are essential for mammary gland development and ductal morphogenesis [66], and that calcium signaling is indispensable for milk secretion during lactation [67].

### 4.3. Candidate Genes Implicated in Lactation Biology

Based on the key KEGG pathways, we identified five genes (COL1A1, PTGES, PRKAG3, PDGFRB, and RYR1) that play important roles in mammary gland development and lactation. COL1A1, which encodes the major structural component of type I collagen, plays an important role in mammary gland development. Type I collagen guides ductal elongation and branching morphogenesis [68] and shows stage-dependent regulation during mammary lactation and involution. Its expression in mammary fibroblasts contributes to stromal remodeling [69], while alterations in collagen I organization influence epithelial patterning and ductal architecture [70]. Together, these studies indicate that COL1A1 is closely associated with mammary gland development and structural remodeling across reproductive stages. PTGES showed consistent upregulation at both the transcriptional (RNA-seq) and epigenetic (H3K4me3) levels. PTGES encodes prostaglandin E synthase, a key enzyme in the arachidonic acid pathway that mediates prostaglandin signaling during mammary development and lactation [71,72]. Importantly, enrichment of H3K4me3 at the PTGES promoter is consistent with its transcriptional activation, as H3K4me3 is a well-characterized histone modification that marks active promoters and facilitates recruitment of transcriptional initiation complexes [73,74]. PRKAG3 encodes the γ3 subunit of AMPK, a central regulator of cellular energy balance [75]. Its downregulation may indicate a shift toward stable energy utilization during peak lactation.

PDGFRB encodes a receptor tyrosine kinase that regulates stromal proliferation, extracellular matrix remodeling, and epithelial–stromal communication—key processes in mammary gland development [76]. PDGFRB-positive fibroblasts contribute to ductal architecture and branching morphogenesis, and transcriptome studies in lactating ruminants have identified PDGFRB as a remodeling-related hub gene across lactation stages [77]. Although direct evidence for its role in milk synthesis is limited, current studies support PDGFRB as an important regulator of the stromal environment required for mammary development and lactational function.

## 5. Conclusions

This study aimed to elucidate the epigenetic and transcriptional mechanisms regulating lactation in Yili horses. Integrated CUT&Tag and RNA-seq revealed lactation-stage-dependent H3K4me3 remodeling and candidate regulatory genes/pathways in Yili horse mammary tissue. These findings provide a resource for follow-up validation and hypothesis-driven functional studies. Translation to breeding applications will require testing candidate DNA variants in larger populations with lactation phenotypes and developing minimally invasive assays (hair follicle/blood). Furthermore, future comparative studies across equine species, such as donkeys, could yield broader insights into the evolution and specialization of lactation mechanisms.

## Figures and Tables

**Table 1:** H3K4me3 CUT&Tag data summary of Yili horse mammary tissue.

Sample	Raw Reads	Clean Reads	Clean Bases	Q30	Mapped Reads	H3K4me3/IgG Ratio
M1_RXL_H3K4me3	30,115,010	29,941,540	4,064,633,901	93.76%	29,806,100 (99.55%)	7.85
M1_RXL_IgG	3,843,304	3,824,328	521,559,374	94.61%	3,796,467 (99.27%)	/
M2_RXL_H3K4me3	35,374,116	35,237,368	4,629,328,937	94.34%	35,086,634 (99.57%)	24.86
M2_RXL_IgG	1,431,056	1,423,980	193,043,293	94.80%	1,411,416 (99.12%)	/
M3_RXL_H3K4me3	34,016,384	33,889,420	4,508,416,020	94.78%	33,758,473 (99.61%)	8.39
M3_RXL_IgG	4,069,608	4,050,768	548,687,607	95.09%	4,022,695 (99.31%)	/
M4_RXL_H3K4me3	24,341,528	24,265,572	3,087,424,096	94.76%	24,179,611 (99.65%)	13.86

Note: Clean reads: Reads that passed the quality filter. Clean bases: The total number of bases in the clean reads. Q30: The percentage of bases in the clean reads that have a Phred quality score ≥ 30.

## Data Availability

The datasets generated and/or analysed during the current study are available in the CNCB-NMDC repository under the accession number PRJCA047836.

## Supplementary Materials

The following supporting information can be downloaded at https://www.mdpi.com/article/10.3390/ani16060891/s1, Table S1: RNA-seq data summary of Yili horse mammary gland tissue; Table S2: Differentially expressed genes (DEGs) between S1 and S2 groups; Table S3: GO enrichment of upregulated differentially expressed genes; Table S4: KEGGE enrichment of upregulated differentially expressed genes; Table S5: Peaks and their annotated genes in group S2; Table S6: Peaks and their annotated genes in group S1; Table S7: KEGG enrichment of H3K4me3 upregulated genes; Table S8: KEGG enrichment of H3K4me3 downregulated genes; Table S9: Upregulated and downregulated motif enrichment analysis; Table S10: Concordantly up- and downregulated H3K4me3 target genes identified by integration of CUT&Tag and RNA-seq; Table S11: Overlap analysis of up/downregulated genes from RNAseq and H3K4me3-enriched regions from CUT&Tag. Figure S1: Genome-wide reads mapping heatmap analysis of control antibody; Figure S2: GO enrichment analysis of the genes shared between the two omics; Figure S3: KEGG pathway enrichment of overlap genes.

## Abbreviations

The following abbreviations are used in this manuscript:

PTGES	Prostaglandin E Synthase
PRKAG3	Protein Kinase AMP-Activated Non-Catalytic Subunit Gamma 3
COL1A1	Collagen, Type I, Alpha 1
PDGFRB	Platelet-Derived Growth Factor Receptor Beta
RYR1	Ryanodine Receptor 1
TFs	Transcription Factors
DEGs	Differentially Expressed Genes
