# Supplementary figures and images for: H3K4me3 CUT&Tag and Transcriptome Analysis Reveal the Epigenetic Regulatory Landscape in Mammary Gland Tissues of Yili Horses at Different Lactation Stages
Source: Animals (Basel). 2026 Mar 12;16(6):891. doi: 10.3390/ani16060891 (PMC13023263; doi:10.3390/ani16060891)

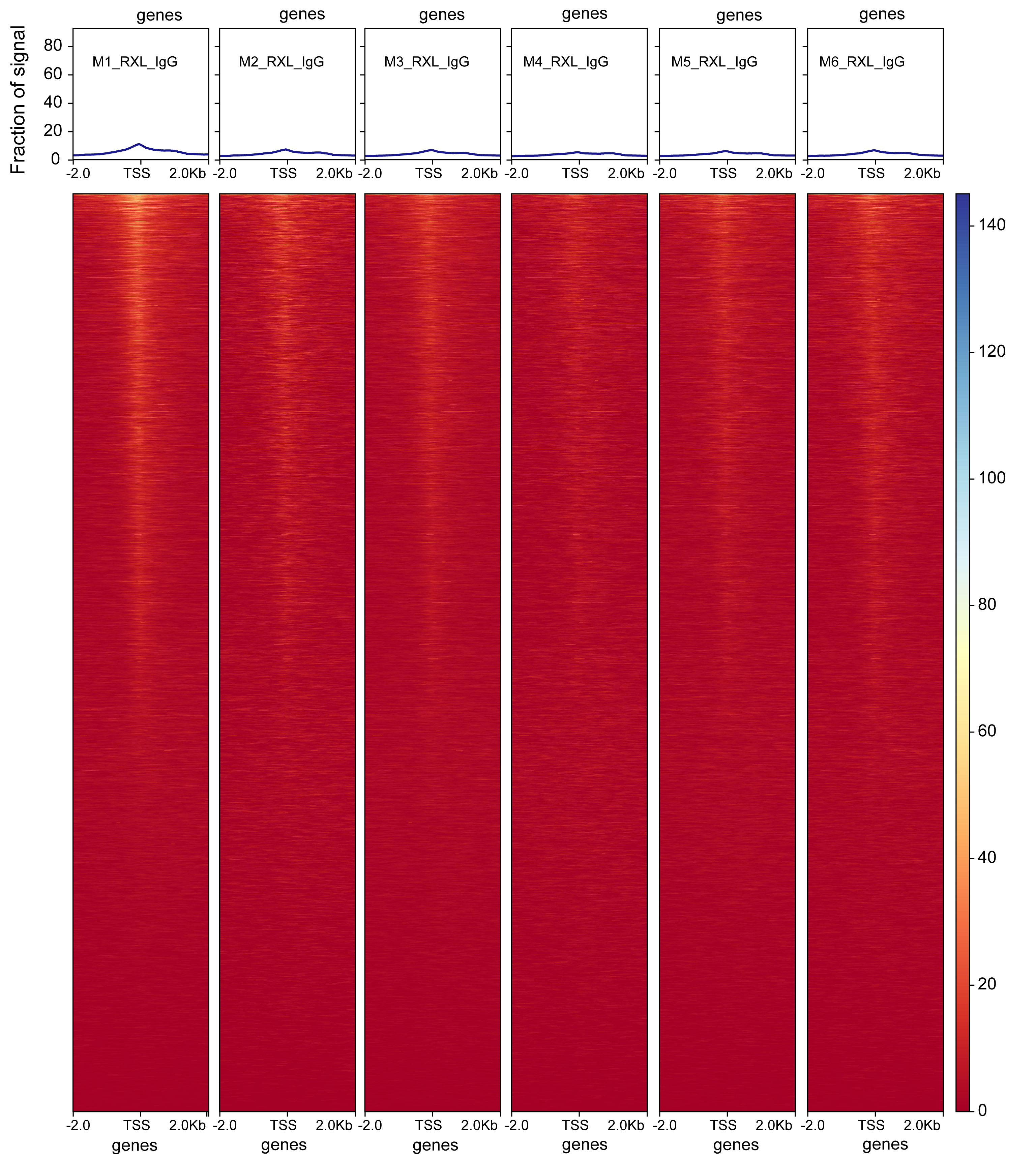

Supplement: Supplementary file 1 [file animals-16-00891-s001.zip › Figure S1 Control antibody mapping heatmap.tif]

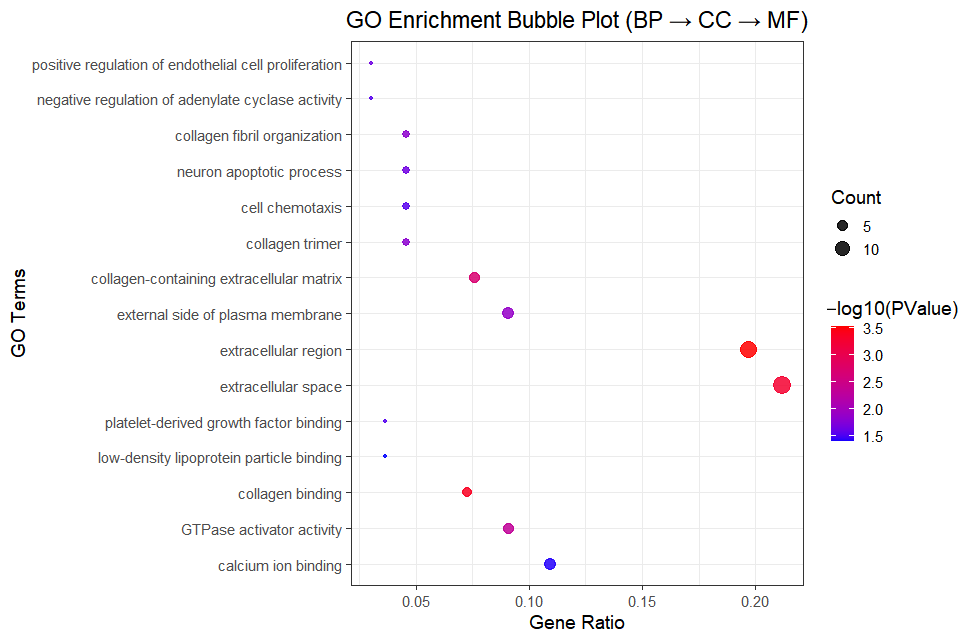

Supplement: Supplementary file 1 [file animals-16-00891-s001.zip › Figure S2 GO enrichment analysis of the genes shared between the two omics.tiff]

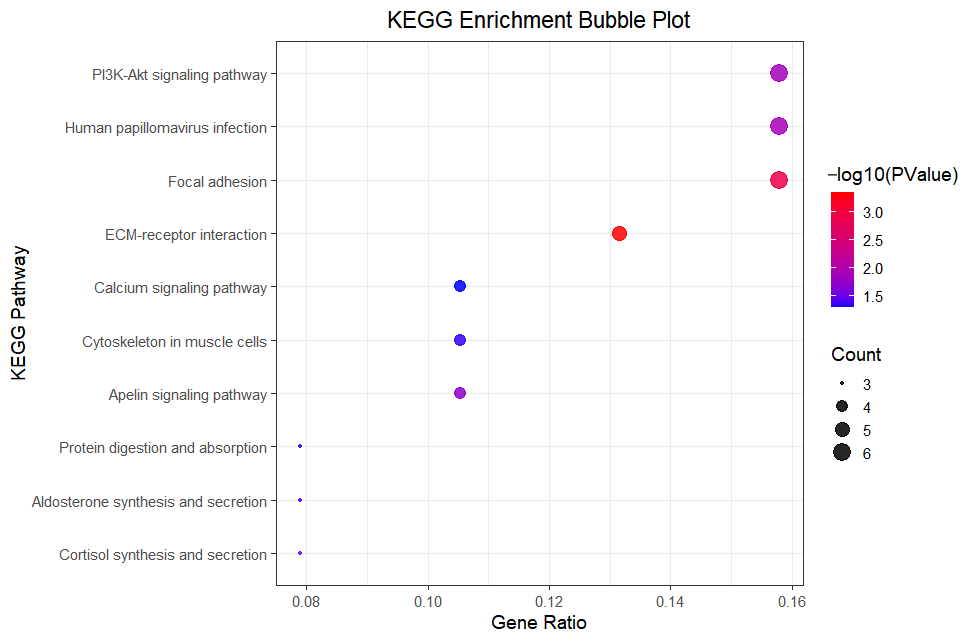

Supplement: Supplementary file 1 [file animals-16-00891-s001.zip › Figure S3 KEGG pathway enrichment of overlap genes.tiff]
